# Hippocampal Connectivity of the Presubiculum in the Common Marmoset (*Callithrix jacchus*)

**DOI:** 10.3389/fncir.2022.863478

**Published:** 2022-07-04

**Authors:** Yoshiko Honda, Tetsuya Shimokawa, Seiji Matsuda, Yasushi Kobayashi, Keiko Moriya-Ito

**Affiliations:** ^1^Department of Anatomy and Neurobiology, School of Medicine, Tokyo Women’s Medical University, Tokyo, Japan; ^2^Division of Anatomy and Embryology, Department of Functional Biomedicine, Ehime University, Toon, Japan; ^3^Department of Anatomy, National Defense Medical College, Tokorozawa, Japan; ^4^Department of Brain Development and Neural Regeneration, Tokyo Metropolitan Institute of Medical Science, Tokyo, Japan

**Keywords:** tracer injection, subiculum, postsubiculum, projection, rostrocaudal topography, entorhinal cortex, memory circuit

## Abstract

The marmoset (a New World monkey) has recently received much attention as an experimental animal model; however, little is known about the connectivity of limbic regions, including cortical and hippocampal memory circuits, in the marmoset. Here, we investigated the neuronal connectivity of the marmoset, especially focusing on the connectivity between the hippocampal formation and the presubiculum, using retrograde and anterograde tracers (cholera toxin-B subunit and biotin dextran amine). We demonstrated the presence of a direct projection from the CA1 pyramidal cell layer to the deep layers of the presubiculum in the marmoset, which was previously identified in the rabbit brain, but not in the rat. We also found that the cells of origin of the subiculo-presubicular projections were localized in the middle part along the superficial-to-deep axis of the pyramidal cell layer of the distal subiculum in the marmoset, which was similar to that in both rats and rabbits. Our results suggest that, compared to the rat and rabbit brains, connections between the hippocampal formation and presubiculum are highly organized and characteristic in the marmoset brain.

## Introduction

Many neurophysiological and behavioral studies have been conducted using rodents, based on the hypothesis that the major circuits of the memory system are evolutionarily conserved across animal species, including primates. Previous studies indicated that the so-called “perforant path” provides a major connectional route from the entorhinal cortex (EC) to the hippocampal formation [i.e., the dentate gyrus (DG), cornu ammonis (CA), and subiculum (Sub)] in many animal species (Witter and Amaral, 1991; van Groen et al., 2002; Witter, 2007). However, the connectivity of parahippocampal areas [i.e., the periallocortical regions including the presubiculum (PreS), parasubiculum (ParS), and EC], especially of the PreS, has not been sufficiently investigated. To elucidate such fundamental connections, including the input and output connections of PreS, we investigated the morphological features of memory circuits, especially between the hippocampus and parahippocampal areas, in rats (*Rodentia*) and rabbits (*Lagomorpha*) using the tracer injection technique. First, we studied hippocampal-parahippocampal connections in rats and provided a comprehensive view of presubiculo-entorhinal projections (Honda and Ishizuka, 2004), intrinsic connections within the PreS (Honda et al., 2008), and entorhino-CA1 and entorhino-subicular projections (Honda et al., 2012). Thereafter, we investigated the hippocampal-parahippocampal connections in rabbits and demonstrated that all the connections stated above were also abundant in rabbits (Honda and Shibata, 2017). Interestingly, several connectional features were different between rats and rabbits; for example, the rabbit PreS directly received abundant inputs from the pyramidal cells of CA1 and had many reciprocal connections with the superficial layers of the EC (Honda and Shibata, 2017). Based on these findings, the hippocampal-parahippocampal connections in primates should be comparatively analyzed. The common marmoset (*Callithrix jacchus*), a New World monkey, has become a key non-human primate model for neuroscience research (Tokuno et al., 2012), especially as a model of age-related neurodegenerative diseases (Okano et al., 2012), including Alzheimer’s disease (Philippens et al., 2017; Sato et al., 2020). However, little is known about the connectivity between the hippocampal and parahippocampal areas of the marmoset. Elucidation of the structural organization of the memory circuit in the normal marmoset brain in comparison with neurodegenerative disease models should contribute to a better understanding of the pathological mechanisms. To address whether basic memory circuits are common among animal species, including rats, rabbits, and marmosets, and whether connections are present that are the characteristic of the marmoset brain, we used retrograde and anterograde tracer injection methods to study the organization of the input and output connections of the hippocampal and parahippocampal areas in the marmoset, especially focusing on the connectivity of the PreS.

## Materials and Methods

All experimental procedures were conducted in common marmoset monkeys (*Callithrix jacchus*) by the guidelines of the National Institutes of Health (United States) for the Use of Laboratory Animals, under a protocol approved by the Animal Care and Use Committee of the Tokyo Metropolitan Institute of Medical Science (TMIMS) and Ehime University. Ten adult marmosets (seven males and three females, aged 2-10 years and weighing 250–454 g) raised in a breeding colony at TMIMS and Ehime University served as animal subjects in this study. Every effort was made to minimize the number of animals used and the pain and distress experienced by the animals. The tracers used were cholera toxin-B subunit (CTB) for retrograde and anterograde labeling and biotinylated dextran amine (BDA) for anterograde labeling. The case details for all animals are presented in Table 1.

**TABLE 1:** Summary of tracer injections described in text.

Case no.	Sex	Age	Tracer	Injection site		
				
				AP (from interaural)	ML	DV (from cortical surface)
09D	m	3y4m	CTB (L)	5.0	–4.5	3.0
09E	m	3y4m	CTB (L)	5.0	–4.5	3.0
			BDA (R)	5.0	4.5	3.0
10F	m	2y6m	CTB+BDA (L)	5.0	–4.5	3.3
13H	m	2y9m	CTB (L)	5.0	–4.5	3.3
			BDA (R)	5.0	4.5	3.5
M23	f	10y5m	CTB (L)	6.0	–4.5	3.0
M25	f	8y11m	CTB (L)	6.0	–4.5	2.8
			BDA (R)	6.0	4.5	2.92
M27	f	9y2m	CTB (L)	6.0	–4.5	2.6
			BDA (R)	6.0	4.5	2.57
M29	m	9y0m	CTB (L)	3.5	–5.5	4.0
08Q	m	3y1m	BDA (R)	6.0	4.5	3.0
08R	m	3y1m	BDA (R)	5.0	4.5	3.5

### Surgery and Tracer Injections

For surgery, each monkey was premedicated with ketamine hydrochloride (10 mg/kg body weight, intramuscular) and sodium pentobarbital (40 mg/kg body weight, intramuscular) for sedation and sodium thiopental (30 mg/kg body weight, intraperitoneal) for anesthesia. Each animal was placed in a stereotaxic frame and a hole was made in the skull at coordinates according to the atlas of Paxinos et al. (2012) (Table 1). For tracer injections, a glass micropipette (inner tip diameter: 30–70 μm) filled with 2% low-salt CTB (List Biological Laboratories, Campbell, CA, United States) dissolved in distilled water or 10% BDA (10,000 MW; Molecular Probes, Eugene, OR, United States) dissolved in saline was stereotaxically lowered through the hole under manipulator guidance. The tracer solution (0.3–0.5 μl) was injected by pressure using a 1-μl Hamilton syringe that was inserted into each glass capillary tube. In all cases except one, CTB was injected into the left hemisphere and BDA was injected into the right hemisphere. In one case (case 10F), a mixed solution of CTB and BDA was injected into the left hemisphere. In four cases, injections were performed bilaterally, whereas, in the other five cases, CTB and/or BDA were injected on only one side (Table 1).

### Fixation and Cutting

After a survival period of 7 days, marmosets were deeply anesthetized with an intramuscular injection of ketamine hydrochloride (10 mg/kg body weight), followed by an intraperitoneal injection of sodium thiopental (30 mg/kg body weight), and perfused transcardially with physiological saline followed by 4% paraformaldehyde in 0.1 M phosphate buffer (PB) at pH 7.4. The brains were extracted and post-fixed in 4% paraformaldehyde in PB overnight, cryoprotected in 20% sucrose or glycerol in PB (4°C), blocked, and cryosectioned in the coronal plane (50-μm thickness). Four parallel series of serial sections were collected in PB, three of which were used in the present study. The first series was histochemically reacted with 3,3’-diaminobenzidine-4HCl (DAB)-nickel as the chromogen, the second series was reacted for BDA by visualization with DAB, and the third was reacted for both BDA (with DAB-nickel) and CTB (with DAB). In some cases, the second series was used for CTB visualization using DAB with biotin blocking (described in the following section).

### CTB Immunohistochemistry

To visualize CTB-labeled cells immunohistochemically, sections were first incubated in Tris-buffered saline (TBS) containing 2% normal rabbit serum and 0.5% Triton X-100 overnight at 4°C, and then, in TBS containing goat anti-CTB (1:30000 or 1:60000; List Biological Laboratories; Table 2), 1% normal rabbit serum, and 0.5% Triton X-100 for 48 h at 4°C. After rinsing in TBS, sections were incubated for 1 h at room temperature in biotinylated rabbit anti-goat IgG (H+L) solution (1:400; Vector Laboratories, Burlingame, CA, United States) containing 1% normal rabbit serum and 0.5% Triton X-100 in TBS. Following a series of rinses in TBS, the sections were incubated in avidin-biotin horseradish peroxidase (HRP) complex solution (Vectastain Elite ABC kit; Vector Laboratories) for 1 h, rinsed in TBS, and treated with nickel-intensified DAB solution containing 0.02% DAB, 0.6% nickel (II) sulfate hexahydrate, and 0.003% H_2_O_2_ in TBS for 30 min. After several washes in TBS, all the sections were mounted onto gelatin-coated slides, dried, and counterstained with thionine. The specimens were then dehydrated in a graded series of alcohol solutions, cleared with xylene, and cover-slipped.

**TABLE 2:** Primary antibody used in this study.

Antibody	Description of immunogen	Source, host species, Cat. #, PRID	Concentration used
Anti-cholera toxin B subunit	Purified choleragenoid, the B subunit of cholera toxin	List Biological Laboratories, goat polyclonal,cat. #703, PRID: AB_10013220	1:30000 or 1:60000

### BDA Histochemistry

To visualize BDA-labeled fibers and terminals, free-floating sections were incubated overnight at 4°C in Tris-HCl buffer (pH 7.6) containing 0.5% Triton X-100 (Sigma, St. Louis, MO, United States), rinsed in TBS, and incubated in TBS containing elite avidin-biotinylated HRP complex (1:200; Vector Laboratories) for 1 h. The sections were rinsed in TBS, treated with TBS containing 0.04% DAB and 0.003% H_2_O_2_ for 30 min, rinsed again in TBS, and mounted on gelatin-coated glass slides. After the sections were dried, they were stained and cover-slipped as described in Section “CTB immunohistochemistry.”

### Biotin Blocking

In two cases (cases M25 and M27), the biotin-blocking method was performed by using the Avidin/Biotin blocking kit (Vector Laboratories) to block BDA labels in the sections, because both BDA and CTB labels were visualized simultaneously by treatment with the avidin-biotin HRP complex and DAB in the same sections, and distinguishing their anterograde labels was difficult. Sections were incubated in TBS containing 2% normal rabbit serum and 0.5% Triton X-100 overnight at 4°C, followed by incubation in avidin D solution for 30 min. After rinsing with TBS, the sections were incubated in a biotin solution for 30 min and rinsed again with TBS. Subsequently, CTB immunohistochemistry was performed as described in the previous section.

### CTB and BDA Visualization

In five cases (cases 09E, 10F, 13H, M25, and M27), to visualize both CTB and BDA in the same section, one series of sections was reacted for BDA with DAB-nickel, followed by CTB visualization with DAB. Sections were incubated overnight at 4°C in Tris-HCl buffer (pH 7.6) containing 0.5% Triton X-100, rinsed in TBS, and incubated in TBS containing elite avidin-biotinylated HRP complex (1:200; Vector Laboratories) for 1 h. Sections were rinsed in TBS and treated with nickel-intensified DAB solution containing 0.02% DAB, 0.6% nickel (II) sulfate hexahydrate, and 0.003% H_2_O_2_ in TBS for 30 min. After several washes in TBS, the sections were incubated in TBS containing 2% normal rabbit serum and 0.5% Triton X-100 overnight at 4°C, and then, in TBS containing goat anti-CTB (1:30000 or 1:60000; List Biological Laboratories; Table 2), 1% normal rabbit serum, and 0.5% Triton X-100 for 48 h at 4°C. After rinsing in TBS, the sections were incubated for 1 h at room temperature in a biotinylated rabbit anti-goat IgG (H+L) solution (1:400; Vector Laboratories) containing 1% normal rabbit serum and 0.5% Triton X-100 in TBS. Following a series of rinses in TBS, the sections were incubated in avidin–HRP complex solution (Vectastain Elite ABC kit; Vector Laboratories) for 1 h, rinsed in TBS, and treated with nickel-intensified DAB solution containing 0.02% DAB and 0.003% H_2_O_2_ in TBS for 30 min. After several washes in TBS, all sections were mounted onto gelatin-coated slides, dried, stained, and cover-slipped, as described in Section “CTB immunohistochemistry.”

### Imaging and Data Analysis

**FIGURE 1:**
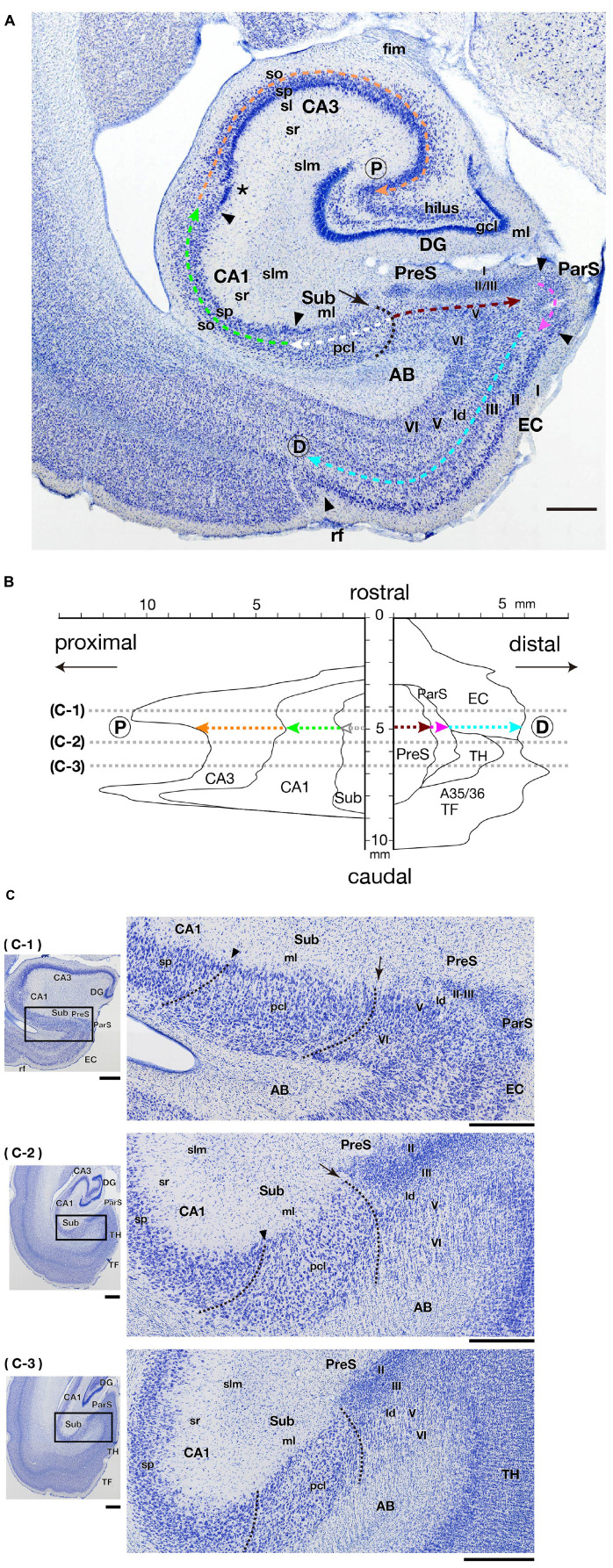
Cytoarchitecture and positional relationships of hippocampal and parahippocampal areas in the marmoset. **(A)** Photomicrograph of a frontal section including the hippocampal formation and some parahippocampal areas (PreS, ParS, and EC). Black arrowheads indicate the boundaries between areas at their surface level (just beneath the molecular layer). The asterisk indicates the position of presumptive CA2. The black dotted line indicates the boundary between Sub and PreS, and the black arrow indicates the origin of the y-axis of the two-dimensional unfolded map in panel **(B)**. Colored dotted arrows indicate the proximodistal extent of each area from the Sub/Pre boundary to the proximal edge of CA3 (as indicated by P within the circle) or to the rhinal fissure, i.e., the distal edge of EC (as indicated by D within the circle). Scale bar = 1 mm. **(B)** An example of a two-dimensional unfolded map of the hippocampal and parahippocampal areas. The Sub/PreS boundary is set as the zero-point of the y-axis, and colored dotted arrows indicate the length of each region within one frontal section that corresponds with **(A)**. Gray dotted lines indicate the rostrocaudal level of the sections shown in panel **(C)**. **(C)** Photomicrographs of frontal sections of three different rostrocaudal levels at an interval of 1.6 mm (between c-1 and c-2) or 1 mm (between c-2 and c-3), as indicated in panel **(B)**. The enlarged photomicrograph of the rectangle in each left panel is shown in the right row. Arrows and arrowheads indicate the surface point of Sub/PreS and CA1/Sub boundary, respectively. Dotted lines indicate the presumptive boundaries between CA1/Sub and Sub/PreS in each section. Scale bars = 1 mm in the left panels and 500 μm in the right panels. A35/36, areas 35 and 36 (the perirhinal cortex); AB, angular bundle; CA, cornu ammonis; D, distal; DG, dentate gyrus; EC, entorhinal cortex; fim, fimbria; ld, lamina dissecans; ml, molecular layer; P, proximal; ParS, parasubiculum; pcl, pyramidal cell layer; PreS, presubiculum; rf, rhinal fissure; slm, stratum lacunosum-moleculare; so, stratum oriens; sp, stratum pyramidale; sr, stratum radiatum; Sub, subiculum; TF, area TF; TH, area TH.

Retrograde and anterograde labels were mapped using a computer-assisted microscope system and data analysis program (Neurolucida™ 11.06, MicroBrightField Inc., Williston, VT, United States). To reconstruct two-dimensional unfolded maps of the hippocampal formation, PreS, ParS, and EC, the transverse (proximodistal) extent of the pyramidal cell layer of CA and Sub, a vertical middle layer of PreS and EC, and layers II-III of the ParS were measured for each section (colored dotted lines in Figure 1A). The lengths of these areas were plotted on a horizontal line, and the Sub/PreS boundary was set as the origin of the proximodistal axis (Figure 1B). Digital photomicrographs of bright-field images were obtained using LINCE™ (Path Imaging, Inc., Tokyo, Japan) in extended focus mode. There were 25 focus levels, and the focus step size was 5 μm. Dark-field images were obtained using a Nikon Eclipse 80i microscope equipped with a Nikon DS-Ri2 digital camera system (Nikon, Tokyo, Japan) at 4,908 × 3,264 pixels.

The captured digital images were trimmed and adjusted to obtain the optimal resolution, brightness, and contrast using Adobe Photoshop™, ver. 23.1.0 (Adobe Systems, Inc., San Jose, CA, United States). Two-dimensional unfolded maps were prepared as previously described (Honda and Ishizuka, 2004), and histograms were created using Microsoft Excel™ 2011 (Microsoft Co., Redmond, WA, United States). All graphs were modified and combined using Adobe Illustrator™, ver. 26.0.2 (Adobe Systems, Inc., San Jose, CA, United States).

## Results

### Nomenclature and Cytoarchitectonic Description

In the present study, we defined the hippocampal formation as DG, CA1-3, and Sub. We also defined the cortical areas adjacent to the hippocampal formation as “parahippocampal areas,” including the PreS, ParS, and EC. Following the marmoset brain atlas (Paxinos et al., 2012), we defined the boundaries between these areas in each section. In the marmoset CA3, the superficial part of the pyramidal cell layer was composed of densely packed cells, whereas in the deeper part, the cells were uniformly distributed (Figure 1A). In contrast, pyramidal cells of marmoset CA1 were present in a broad layer of uniformly distributed cells. A narrow region (proximodistally 400–500 μm in width) adjacent to the CA3/CA1 boundary was likely to correspond to the CA2 region (asterisk in Figure 1A), but CA2 was not defined because CA2-specific staining was not performed in this study. The CA1/Sub boundary was unclear, but in the middle-to-deep part of the pyramidal cell layer of the Sub, the cell size was slightly smaller, and the distribution density was relatively lower than that in the pyramidal cell layer of CA1 (Figure 1C). The Sub/PreS boundary was defined by the proximal edge of densely packed clusters of small cells in the superficial layers (layers II-III) of PreS (Figures 1A,C). In Nissl-stained sections, the boundary between layers II and III of PreS was unclear (Figures 1A,C); therefore, we did not set a boundary line between them in the present study.

Although a previous study subdivided marmoset EC into six fields (de Góis Morais et al., 2020), the boundary between such subsegments of EC was not identified by Nissl staining alone. Thus, in the present study, EC was defined as a cortical region that was identified by a distinct layer II that was composed of densely stained, large-sized cells (Figure 1A), and we did not parcellate EC into medial (MEC) and lateral (LEC) parts.

### CTB Injection

#### CTB Injection in the PreS

##### Labeled Cells in the Hippocampal Cornu Ammonis Region

**FIGURE 2:**
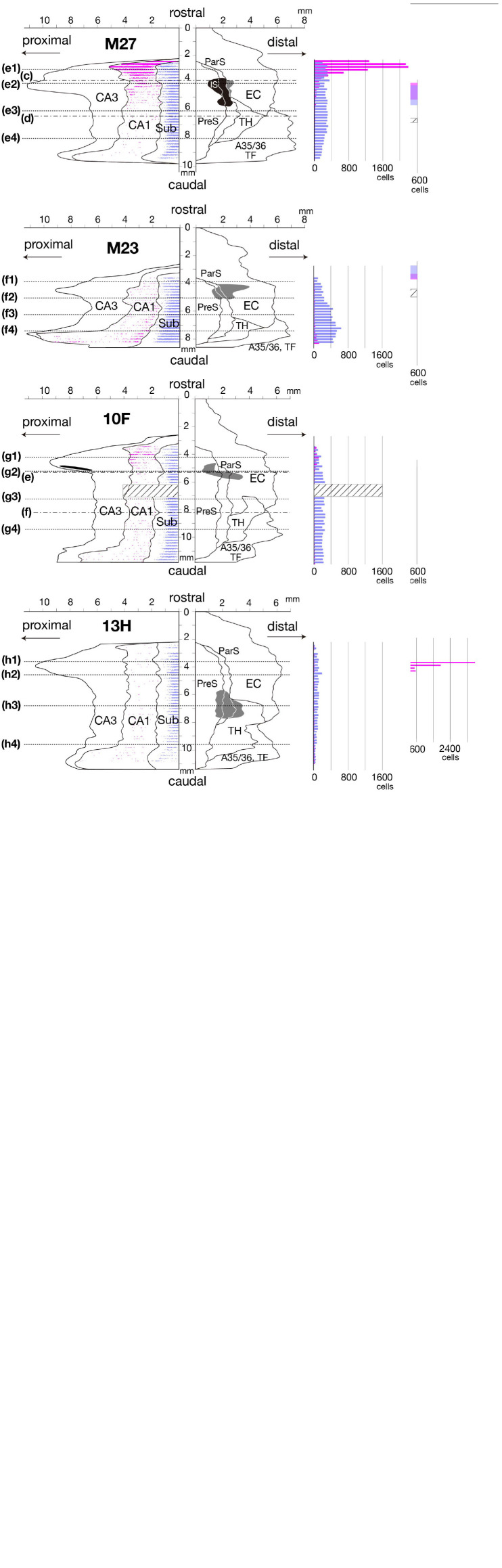
Distributions of CTB injection sites and density of distributions of retrogradely labeled cells in CA1 and Sub are represented on a two-dimensional unfolded map and histogram of each case. Left unfolded maps: Black regions indicate CTB injection sites that extended to all layers. The gray regions in cases 09E, M27, M23, 10F, and 13H indicate the injection sites that was restricted to the superficial layers, and the shaded-gray region in case 09D indicates the injection site of EC that was restricted to the deep layers. Magenta- and blue-filled regions in cases M25 and 09D indicate the portions that overlapped with the center of diffusion of the injection site and plotting of the labeled cells was partially difficult within these regions. Magenta dots and blue dots indicate the distribution of single retrogradely labeled cells in CA1 and Sub, respectively. Dotted lines of each unfolded map indicate the rostrocaudal level of each section in Figure 3. Alternate long-and-short dash lines in the unfolded map of cases M29, M27, and 10F indicate the rostrocaudal level of each panel in Figure 4. Areas of shaded lines in cases M25, 09D, and 10F indicate the rostrocaudal levels of sections with lost parts. IS, injection site. Right histograms: Labeled-cell numbers in the pyramidal cell layer of CA1 (magenta bar) and Sub (blue bar) of each brain section are rostrocaudally aligned along the same y-axis as unfolded map. Magenta and blue cubes indicate the portions that overlapped with the center of diffusion of the injection site, and thus, accurate counting of the cell number was partially difficult within these portions. The bar graphs within cubes represent the number of cells that could be counted. Cubes of shaded lines in cases M25, 09D, and 10F indicate the rostrocaudal levels of sections with lost parts. A35/36, areas 35 and 36 (the perirhinal cortex); CA, cornu ammonis; DG, dentate gyrus; EC, entorhinal cortex; IS, injection site; ParS, parasubiculum; PreS, presubiculum; Sub, subiculum; TF, area TF; TH, area TH. Note that many CA1 cells were retrogradely labeled along the rostrocaudal axis in the experimental cases where CTB injection extended to all layers of PreS (cases M25, 09D, 09E, M29, and M27). On the other hand, fewer labeled cells were observed in CA1 in the cases where CTB injection did not extend to the deep layers of PreS (cases M23, 10F, and 13H). Note also that a large number of labeled cells were distributed in Sub along the rostrocaudal axis in all PreS injection cases.

**FIGURE 3:**
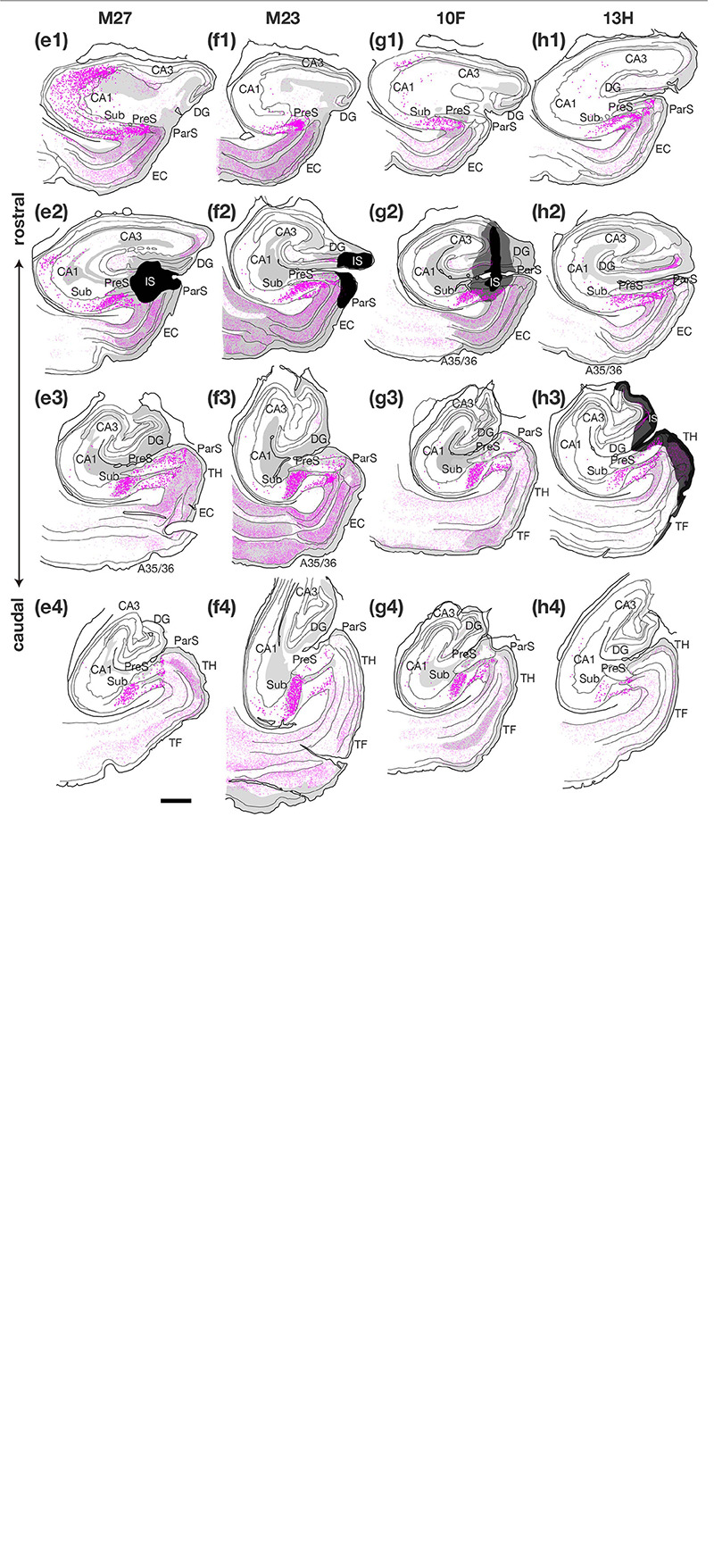
Plotting of retrogradely labeled cells in the hippocampal and parahippocampal areas on the ipsilateral side of the injection site of CTB. Sections in each case are presented from rostral (1) to caudal (4) levels at 1- to 3-mm intervals as indicated in the unfolded maps in Figure 2. Dark magenta dots represent single retrogradely labeled cells in CA1, Sub and PreS, and pale magenta dots indicate single labeled cells in the other regions. Injection sites and their diffusion are indicated by black and dark gray portions, respectively. Regions of anterograde label distribution are represented by light gray portions. Scale bar = 1 mm. A35/36, areas 35 and 36 (the perirhinal cortex); CA, cornu ammonis; DG, dentate gyrus; EC, entorhinal cortex; IS, injection site; ParS, parasubiculum; PreS, presubiculum; Sub, subiculum; TF, area TF; TH, area TH. Note that many labeled cells were distributed along the rostrocaudal axis in the cases where CTB injection extended to all layers of PreS (cases M25, 09D, 09E, M29, and M27). On the other hand, fewer labeled cells were observed in CA1 in the cases where CTB injection did not extend to the deep layers of PreS (cases M23, 10F, and 13H). Note also that many labeled cells were distributed in Sub and PreS along the rostrocaudal axis in all cases.

**FIGURE 4:**
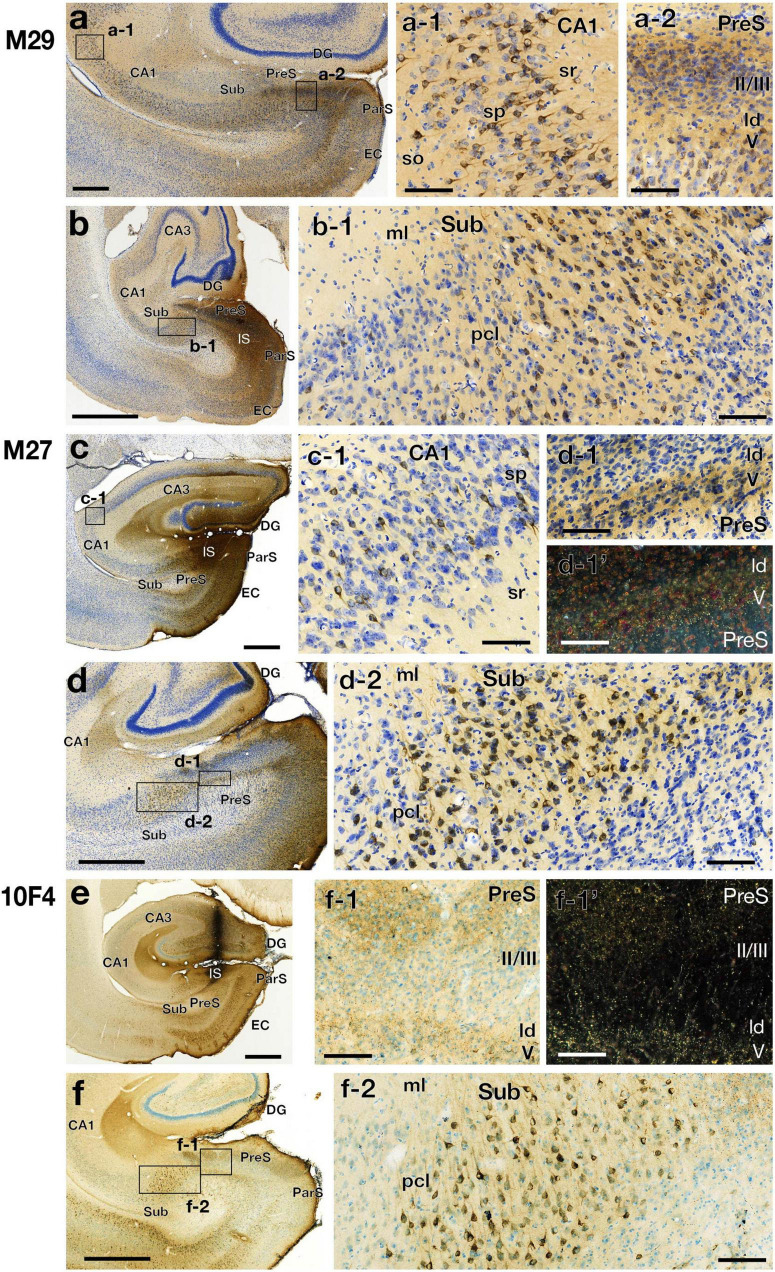
Photomicrographs of the hippocampal and parahippocampal areas in three CTB injection cases (cases M29, M27, and 10F4) on the same side of the injection (left hemisphere). High-magnification images of rectangles in each panel of the left row (a, b, c, d, and f) are shown in the middle and right rows. Panels d-1’ and f-1’ are dark-field images of the same section as d-1 and f-1, respectively. Scale bar = 1 mm in the left row (a-f) and 100 μm in the other panels. CA, cornu ammonis; DG, dentate gyrus; EC, entorhinal cortex; IS, injection site; ld, lamina dissecans; ml, molecular layer; ParS, parasubiculum; pcl, pyramidal cell layer; PreS, presubiculum; so, stratum oriens; sp, stratum pyramidale; sr, stratum radiatum; Sub, subiculum. Note that the labeled CA1 cells were diffusely distributed in the pyramidal cell layer. Note also that, in Sub, labeled cells were densely distributed in the middle part of the pyramidal cell layer, while very few in superficial and deep parts. In PreS, many anterograde labels were observed in layers I, II, and V along with ld.

In the group of experiments with CTB injection where the injection site included all layers of PreS (cases M25, 09D, 09E, M29, and M27 in Table 3), there were numerous retrogradely labeled cells along the longitudinal (rostrocaudal) axis of the ipsilateral CA1 pyramidal cell layer (Figures 2, 3). The labeled cells were diffusely distributed throughout the superficial-to-deep parts of the CA1 pyramidal cell layer (Figures 3, 4). On the other hand, the number of labeled CA1 cells was much lower in cases with injections mostly confined to the superficial layers (layers I-III) of PreS and did not extend to the deep layers (layers V-VI) (cases M23, 10F, and 13H in Figures 2,3 and Table 3). These results suggest a large number of projections from the pyramidal cell layer of CA1 to the predominantly deep layers of PreS and fewer projections from the superficial layers.

**TABLE 3:** CTB injection sites and relative amount of tracer taken up in the hippocampal and parahippocampal areas of each experimental case.

	CA3	CA1	Sub	PreS		ParS		EC		TH
						
				sup	deep	sup	deep	sup	deep	
M25	+	+	+	++	++	–	–	–	–	–
09D	–	+	–	+	+	–	–	–	+	–
09E	–	–	–	++	++	++	+	–	–	–
M29	–	–	–	+	++	–	–	–	–	–
M27	–	–	–	++	+	+	+	+	–	–
M23	–	–	–	+	–	+	–	+ (I-II)	–	–
10F	+	–	–	+	–	+	–	+	–	–
13H	–	–	–	+	–	++	–	–	–	+ (sup)

*Volume of CTB uptake: –, no injection; ±, low; +, moderate; ++, high. Abbreviations: CA, cornu ammonis; deep, deep layers (layers V-VI); CTB, cholera toxin B subunit; EC, entorhinal cortex; ParS, parasubiculum; PreS, presubiculum; Sub, subiculum; sup, superficial layers (layers I-III); TH, area TH.*

In case M27, in which the injection site was confined to the mid-to-rostral part of PreS, without including the rostral edge of PreS, the density of retrogradely labeled cells was significantly higher in the most rostral part of CA1 (Figures 2, 3). In cases 09E and M29, where the injection site was in the mid-to-caudal part of PreS, labeled cells were abundant in the rostral half of CA1, with a peak at the mid-rostral level, and the density of labeled cells was relatively low in the caudal region (as seen in the histogram in Figure 2). Thus, cells in the rostral part of CA1 tend to project to a wide range of PreS regions along the rostrocaudal axis.

Of the cases in which the injection site included all layers of the PreS, the injection sites in cases 09E, M29, and M27 were confined to the mid-to-distal portion, without including the proximal edge of the PreS (Figure 2). In all three cases, the labeled CA1 cells were almost entirely distributed in the proximodistal direction, and there was no significant topographic organization in the distribution pattern of labeled cells in CA1 (Figures 2, 3), suggesting a proximodistally wide range of projections from the local CA1 to the PreS.

##### Labeled Cells in the Sub

**FIGURE 5:**
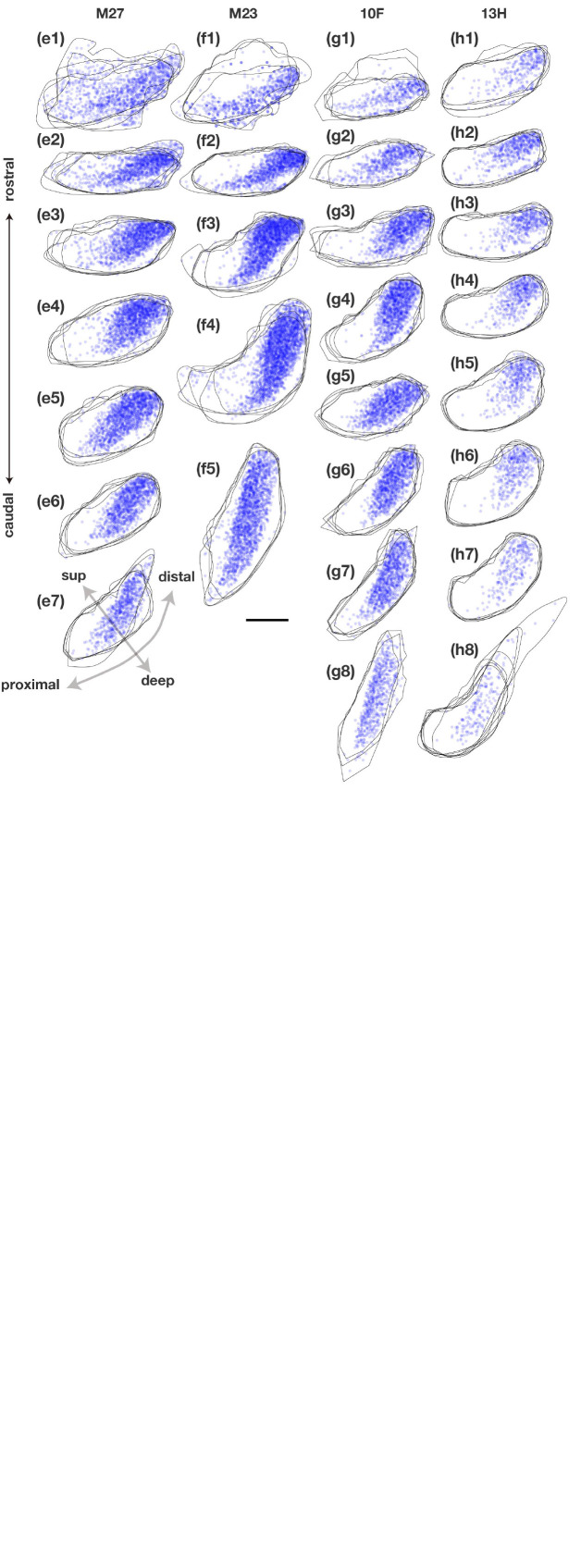
Distribution and density of retrogradely labeled cells in the pyramidal cell layer of Sub on the ipsilateral side of the CTB injection site in each case. Plots of labeled subicular cells with the contour of Sub of five sections (about 1 mm) are superimposed and arranged in a row along the rostrocaudal axis in each case. Pale blue dots represent single retrogradely labeled cells and when they overlap (i.e., the density of labeled cells is increased), the color becomes darker. Injection sites and their diffusion are indicated by dark blue portions and plotting of the labeled cells was partially difficult within these regions. sup, superficial. Scale bar = 500 μm. In most cases, labeled cells were densely localized in the middle superficial-to-deep part of the distal half of Sub at all levels throughout the rostrocaudal axis. In case M25, which injection site included Sub, labeled cells were concentrated also in the proximal-superficial part of Sub.

In all PreS injection cases, a large number of labeled cells were observed in a wide range along the rostrocaudal axis of the pyramidal cell layer of the Sub (Figures 2, 3). In particular, in all cases in which the injection site included the PreS but did not extend to the Sub (cases 09D, 09E, M23, M27, M29, 10F, and 13H in Figure 2 and Table 3), labeled cells were densely distributed in the middle superficial-to-deep part of the distal half of the Sub (near the PreS), whereas fewer were found in the most superficial (just beneath the molecular layer) and deepest parts (adjacent to the white matter) of the pyramidal cell layer of the Sub (Figures 3–5). These results indicate that the presubicular projecting cells are mostly distributed in the vertical middle region of the pyramidal cell layer of the distal Sub in a wide range along the rostrocaudal axis. In all cases, a small number of unlabelled cells were scattered among the labeled-cell clusters of the Sub (Figure 4). In case 10F, in which the injection was confined to the superficial layers of PreS and did not extend to the deep layers (Figure 2 and Table 3), labeled cells were also clustered in the middle superficial-to-deep region of the distal Sub (Figures 4, 5), suggesting that at least the superficial layers of the PreS could be innervated by the Sub. In cases where the injection was mostly confined to the superficial layers of the PreS and did not extend to the deep layers (cases M23, 10F, and 13H), the labeled-cell population was compactly concentrated in the middle superficial-to-deep part of the distal half of the Sub (Figure 5). On the other hand, in case M29, a case of CTB injection confined to the PreS (the injection site covered almost the entire layer of the PreS), labeled cells in the Sub were scattered in the superficial and deep parts other than the middle part. This suggests that the distribution of subicular cells projecting to the deep layers of the PreS seemed to be more diffuse than that of the subicular cells, which innervate the superficial layers of the PreS in the pyramidal cell layer.

#### CTB Injection in the Sub, ParS, and/or Other Cortical Areas (I.e., the Entorhinal Cortex or Area TH)

##### Labeled Cells in the Sub

In case M25, in which the injection site included a rostral portion of the Sub (Figure 2 and Table 3), the labeled cells were widely distributed across the pyramidal cell layer along the proximodistal and superficial-to-deep axes (Figures 3, 5). In this case, the density of labeled cells was higher in the proximal-superficial region, not only in the vertically middle region of the pyramidal cell layer of the Sub (case M25 in Figure 5), but in a wide range along the rostrocaudal axis (Figures 2, 3). These results suggest that both CA1 projection neurons and intrinsic projection neurons are distributed throughout the entire range of the Sub.

##### Labeled Cells in CA1

Compared with case M25 (which injection site included CA1 and the Sub other than the PreS) and case 09D (which injection site included CA1 and EC other than the PreS), the number of labeled cells in CA1 was much lower in cases M23 and 10F (which injection sites extended to the superficial layers of the ParS and EC) and also in case 13H (which injection site extended to the superficial layers of the ParS and area TH) (Figure 4). These results suggest the existence of intrinsic projections within CA1 and direct projections from CA1 to the Sub and also from CA1 to the deep layers of the ParS and/or EC; however, we could not identify the distribution of cells of origins of those direct projections, because we did not have cases of CTB injection confined to the Sub, ParS, EC, or area TH.

#### Distribution of Anterograde Labels in CTB Injection Cases

In the present study, the distribution of anterograde labels in each CTB injection case was also investigated, and we found that it was generally consistent with the results of the BDA injection cases, as described in the following section. In all cases, in which the injection sites included the deep layers of the PreS (cases M25, M29, 09D, 09E, and M27 in Table 3), anterograde labeling was observed in all layers of CA1, including the stratum lacunosum moleculare (Figure 3). Moreover, in all cases in which the injection sites included superficial and/or deep layers of the PreS, anterogradely labeled terminals were densely distributed in layer I, presumed layer II (the superficial part of layers II/III), lamina dissecans, and layer V (Figures 3, 4).

### BDA Injection

#### BDA Injection in the Hippocampal CA Regions

##### Labeled Terminals in the PreS

**FIGURE 6:**
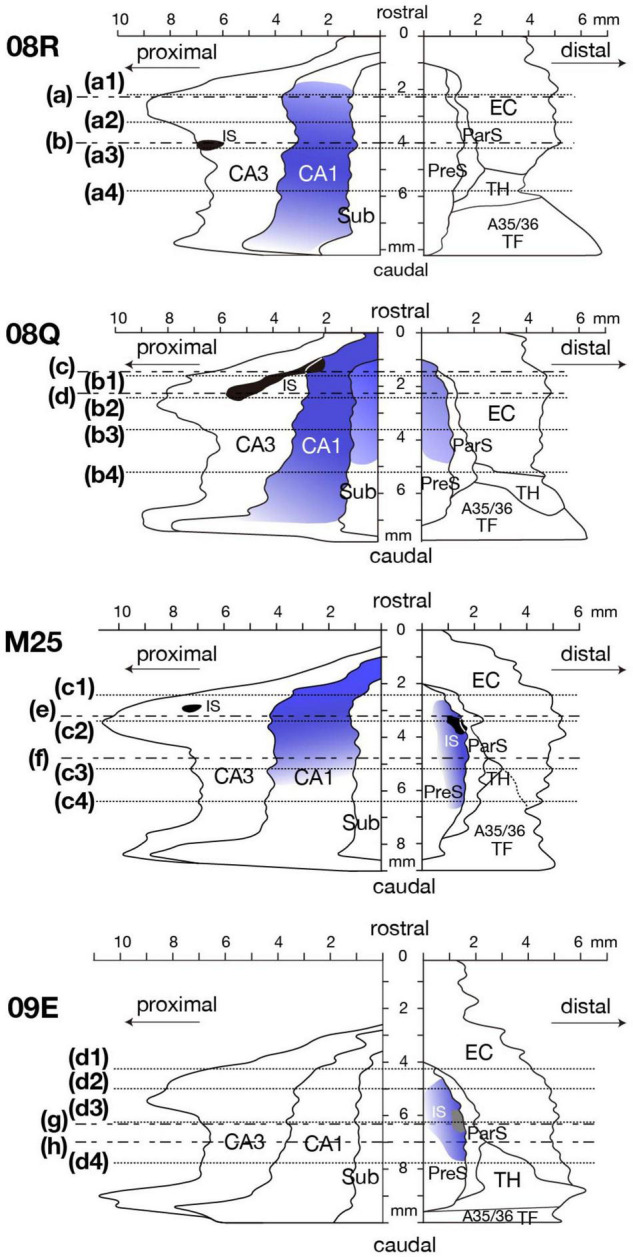
Distribution of BDA injection sites and anterogradely labeled terminals of four cases are represented on a two-dimensional unfolded map. Black regions indicate BDA injection sites that extended to all layers. The gray region in case 09E indicates the injection site that was restricted to the superficial layers of PreS. Each blue colored region indicates the distribution and intensity of labeled terminals. Dotted lines of each unfolded map indicate the rostrocaudal level of each section in Figure 8. Alternate long-and-short dash lines in the unfolded map of cases 08R, 08Q, M25 and 09E indicate the rostrocaudal level of each panel in Figure 7. A35/36, areas 35 and 36 (the perirhinal cortex); CA, cornu ammonis; DG, dentate gyrus; EC, entorhinal cortex; IS, injection site; ParS, parasubiculum; PreS, presubiculum; Sub, subiculum; TF, area TF; TH, area TH. Note that anterograde labels were densely distributed in PreS along the rostrocaudal axis in the experimental cases where BDA injection included CA1 (case 08Q) or the superficial layers of PreS (case 09E) or all layers of PreS (case M25). Note also that many labeled terminals were observed in CA1 in the cases where BDA injection site included CA3 (cases 08R, and M25). In case 08Q, where BDA injection included CA1, labeled terminals were densely distributed also in Sub.

**FIGURE 7:**
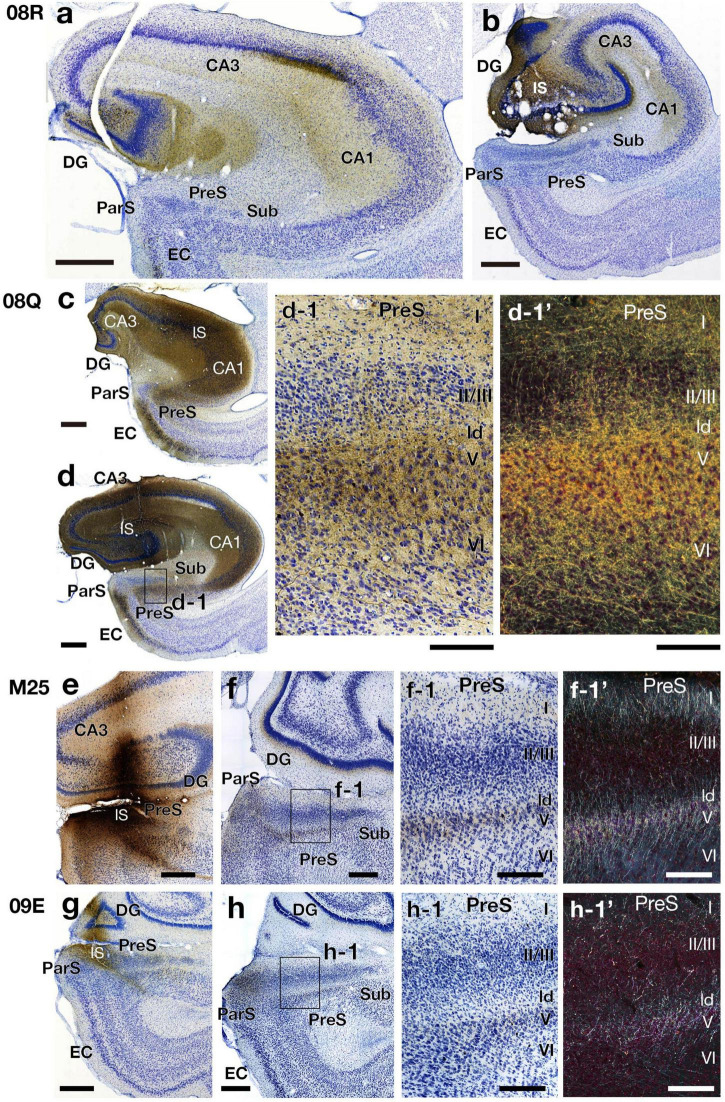
Photomicrographs of the hippocampal and parahippocampal areas in four BDA injection cases (cases 08R, 08Q, M25, and 09E) on the same side of the injection (right hemisphere). High-magnification images of rectangles in d, f, and h are represented in d-1, f-1, and h-1, respectively. Panels d-1’, f-1’ and h-1’ are dark-field images of the same section as d-1, f-1 and h-1, respectively. Scale bar = 200 μm in panels a, b, c, d, f-1, f-1’, h-1 and h-1’, 500 μm in e, f, g, and h and 50 μm in d-1 and d-1’. CA, cornu ammonis; DG, dentate gyrus; EC, entorhinal cortex; IS, injection site; ld, lamina dissecans; ParS, parasubiculum; PreS, presubiculum; Sub, subiculum. Note that, the BDA injection site included CA1 in case 08Q but not included in case 08R. In case 08Q, many fibers and terminal boutons were labeled in layers I, V and VI along with ld in PreS. On the other hand, in case 08R, there were almost no labels in PreS. Also note that in cases M25 and 09E, which injection sites contained a part of PreS, labeled fibers and terminals were observed in layers I, II, and V along with ld in PreS.

**FIGURE 8:**
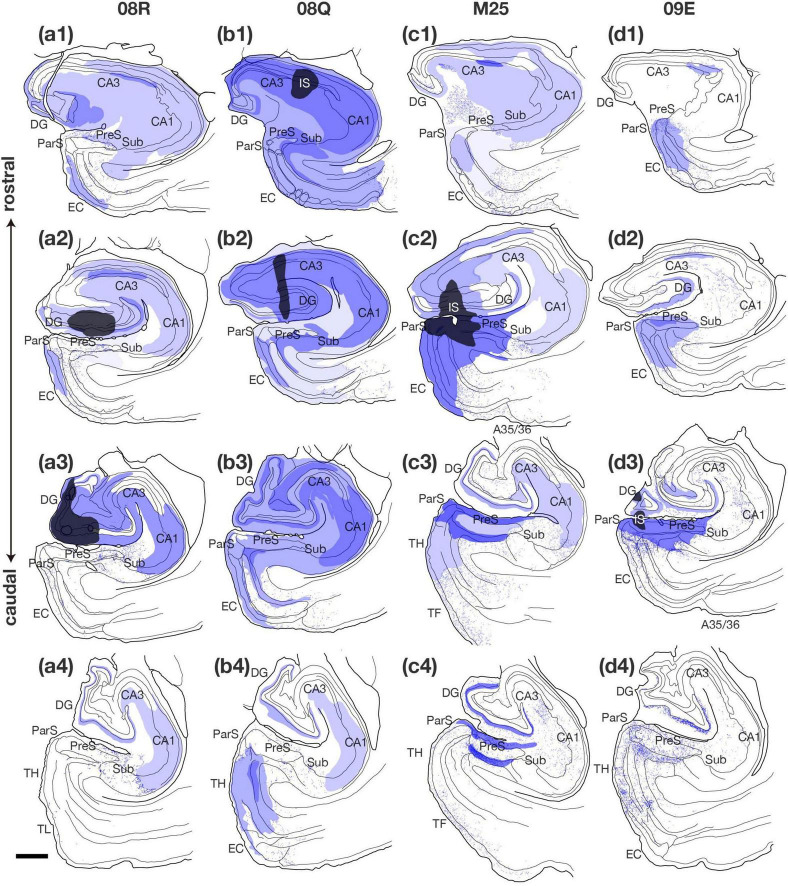
Plotting of anterogradely labeled terminals in the hippocampal and parahippocampal areas on the ipsilateral side of the BDA injection site. Sections in each case are presented from rostral (1) to caudal (4) levels at 1- to 2-mm intervals as indicated in the unfolded maps in Figure 6. Injection sites and their diffusion are indicated by black and dark blue areas, respectively. Blue dots represent single anterogradely labeled terminal boutons. Regions of dense anterograde label distribution are represented by deep or pale purple color and plotting of the labeled termimal boutons was difficult within these regions. Scale bar = 1 mm. A35/36, areas 35 and 36 (the perirhinal cortex); CA, cornu ammonis; DG, dentate gyrus; EC, entorhinal cortex; IS, injection site; ParS, parasubiculum; PreS, presubiculum; Sub, subiculum; TF, area TF; TH, area TH. Note that, in case 08Q, BDA injection site included CA1 and there was a larger number of labeled fibers and terminal boutons in PreS than in case 08R. Note also that, in cases M25 and 09E, which injection sites contained a part of PreS, two parallel bands of anterograde labels (correspond to layers I, II, ld, and layer V) were observed in PreS along the rostrocaudal axis.

Comparing case 08Q (the injection site included a small portion of CA1) with case 08R (the injection site did not include CA1) (Figure 6 and Table 4), the number and density of labeled terminals in the PreS were distinctly different; that is, the density of anterograde labeling was significantly higher in the deep layers of the PreS in case 08Q, whereas very few labeled cells were present throughout all layers of the PreS in case 08R (Figures 7, 8). These results confirm the findings of the CTB injection, in which a large number of projections from CA1 to the deep layers of the PreS were observed.

**TABLE 4:** BDA injection sites and relative amount of tracer taken up in the hippocampal and parahippocampal areas of each experimental case.

	CA3	CA1	Sub	PreS		ParS		EC	
						
				sup	deep	sup	deep	sup	deep
08R	+	–	–	–	–	–	–	–	–
08Q	++	+	–	–	–	–	–	–	–
M25	+	–	–	++	+	±	±	–	–
09E	–	–	–	+	–	–	–	–	–

*Volume of BDA uptake: –, no injection; ±, low; +, moderate; ++, high. Abbreviations: BDA, biotinylated dextran amine; CA, cornu ammonis; deep, deep layers (layers V-VI); EC, entorhinal cortex; ParS, parasubiculum; PreS, presubiculum; Sub, subiculum; sup, superficial layers (layers I-III).*

Although the injection site in case 08Q was a very small and localized area in the proximal portion of the rostral CA1 (without including the rostral edge of CA1), labeled terminals in the PreS were widely distributed along its rostrocaudal and proximodistal axes, and the density was higher in the rostral half (including the rostral part) (Figures 6, 8). This result suggests that there are projections from a small and localized area of CA1 to a broad region of the PreS, at least more than half of the longitudinal axis, including the rostral edge.

##### Labeled Terminals in CA1

In case 09E, in which the injection site did not include the hippocampal CA regions or the deep layers of the PreS, a small number of anterograde labeling was observed in CA1 and the Sub. On the other hand, in cases 08R, M25 (injection sites included CA3), and 08Q (injection sites included both CA1 and CA3), many labeled terminals were observed in CA1. These results suggest that terminations of the CA3-CA1 projection and/or intrinsic projection within CA1 could be involved in the labeled terminals observed in these cases. In case 08R, a small injection site was localized in the proximal edge of the middle-rostrocaudal level of CA3 (Figure 6), and the labeled terminals were distributed throughout the entire CA1 region except for the rostral and caudal edges and were also distributed throughout the whole proximodistal range of CA1, suggesting the existence of a widely diffused innervation from a small part of CA3 to the entire CA1 region.

#### BDA Injection in the PreS

##### Labeled Terminals in CA1

In case 09E, in which the injection sites did not contain CA regions, Sub, ParS, or EC but included a portion of the superficial layers of the PreS (Figure 6 and Table 4), only a small volume of BDA-labeled terminals were observed in all layers of CA1 (Figure 8). On the other hand, in case M25 (the injection site included both superficial and deep layers of the PreS), a large number of anterograde labeling was observed in all layers of CA1, including the stratum lacunosum moleculare (Figure 8). These observations suggest that CA1 projections primarily originate from the deep layers of the PreS, if they exist.

##### Labeled Terminals in the Sub

In case 09E, in which the injection site involved only the superficial layers of the PreS (Figure 6 and Table 4), only a small number of labeled terminals were found in the molecular layer of the Sub (Figure 8). This result suggests that the Sub receives very few projections from the superficial layers of the PreS.

##### Labeled Terminals in the PreS

A large number of labeled terminals were observed in the PreS in cases M25 and 09E, in which the injection sites contained a part of the PreS (Figure 6 and Table 4). The distribution of labeling was concentrated in layer I, presumed layer II (the superficial part of layers II/III), lamina dissecans, and layer V; therefore, two parallel bands of labels seemed to run along the proximodistal axis of the PreS, as seen in Figures 7, 8. These results suggest that layers I-II, lamina dissecans, and layer V mainly participate in the intrinsic connections within the PreS. In case 09E, although the injection site was confined to a small part of the superficial layers of the distal PreS, many labeled terminals were observed in the PreS throughout all rostrocaudal levels (Figures 6, 8). This suggests that the superficial layers of the PreS include intrinsic projection cells that innervate widely along the rostrocaudal axis.

## Discussion

Our study demonstrates that one of the major hippocampal connections, i.e., projections from the Sub to the PreS, is present in the marmoset brain, as well as in rats (Honda and Ishizuka, 2015) and rabbits (Honda and Shibata, 2017). Moreover, our results suggest the presence of an additional projection that is sparse in the rat brain, that is, the projections from the pyramidal cell layer of CA1 to the deep layers of the PreS. These are considered major projections in the marmoset brain and resemble those of the rabbit brain (Honda and Shibata, 2017).

### Connectivity Between CA1 and the PreS

**FIGURE 9:**
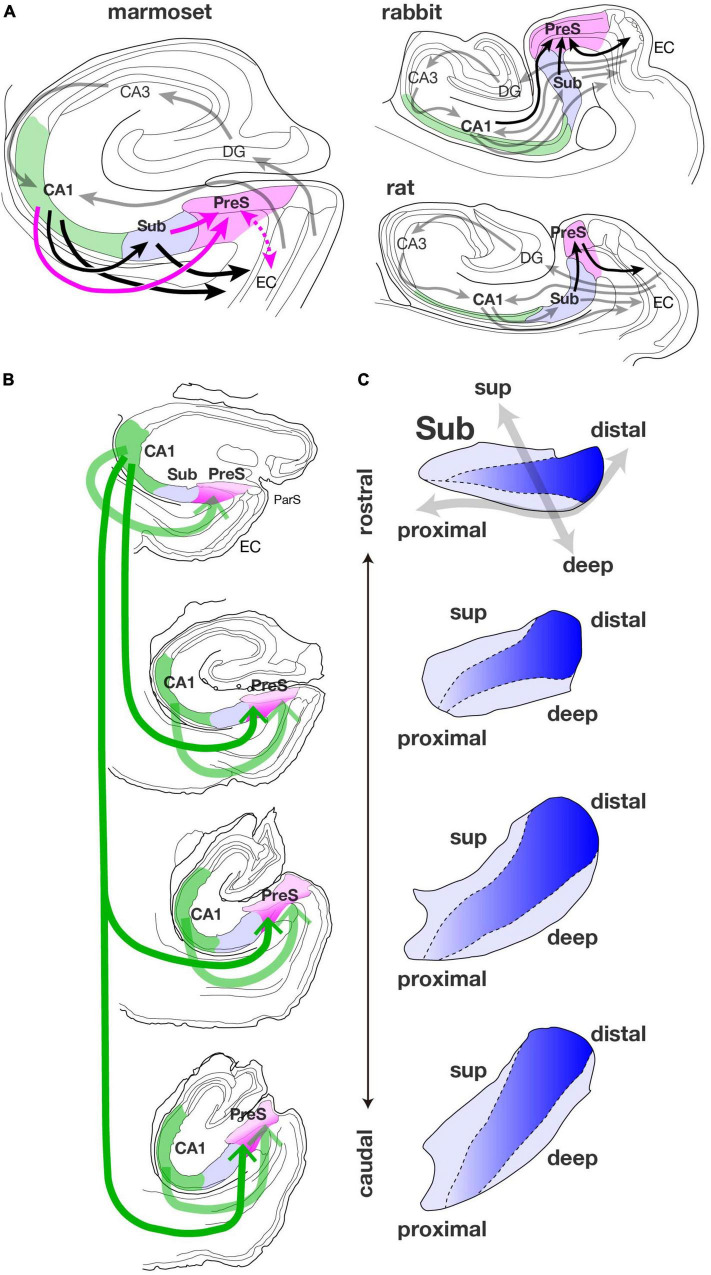
**(A)** Summary diagrams of connections among the hippocampal and parahippocampal areas of the marmoset, rabbit, and rat. In the diagram of the marmoset, magenta arrows indicate projections that were found by our studies. A dotted magenta arrow represents the bidirectional connections between PreS and EC which is currently under investigation. Black allows along with the solid magenta arrows indicate back pathways from CA1 (green-filled area) to EC via Sub (blue-filled area) and/or PreS (magenta-filled area). Gray arrows represent the hippocampal afferents originating from EC and information flow between DG and CA regions, that are conserved from rodents to primates. In the diagrams of rabbit and rat, gray arrows indicate the information flow between EC and the hippocampal formation, and black arrows indicate indirect hippocampal-entorhinal back pathways via PreS. To avoid complication, the projections from EC to CA3 and from EC to Sub are not displayed in these diagrams. **(B)** Schematic diagram showing the spread of CA1-PreS projection along the longitudinal (rostrocaudal) axis of the hippocampal formation. The thickness of green arrow indicates the amount of projection, and the dark magenta area of PreS represents high density of terminal distribution. **(C)** Schematic representation of the localization of PreS projection cells in the pyramidal cell layer of Sub along the longitudinal (rostrocaudal) axis. Blue gradient indicates the population density of PreS projection cells along the proximodistal axis. Note that the dark blue region is distributed in the middle superficial-to-deep part of the distal half of Sub.

Our results indicate that marmoset CA1 contains a large number of presubicular projection cells, similar to those in the rabbit brain (Honda and Shibata, 2017). This suggests that multiple indirect hippocampo-entorhinal back pathways are present in marmosets, including CA1-PreS-EC other than CA1-Sub-EC (Figure 9A). Although projection from CA1 to the PreS has been reported in the rat at the individual cell level (Arszovszki et al., 2014), no significant labeling of CA1 was detected in our previous studies by using HRP-conjugated to wheat germ agglutinin (WGA-HRP), which was injected into various parts of the rat PreS. It is possible that during the evolution of the cerebral cortex, information processing became more complex due to an increase in the amount of information transmitted through CA1 (Rogers Flattery et al., 2020), and as a result, the CA1-PreS projections, which was minor in rodents, become more major in primates. To our knowledge, a direct projection from CA1 to the PreS has not been reported in other primate species, and this is the first time that major CA1-PreS connectivity has been detected in a non-human primate. Since the 1980s, there have been many reports of hippocampal connectivity of monkeys using tracers including WGA-HRP, but some do not recognize the PreS as an independent cortical area but combine it with the Sub and ParS as a “subicular complex.” It is possible that projections from CA1 to the PreS were overlooked because of the large number of projections from CA1 to the Sub.

In rabbits, CA1-PreS connectivity is mostly unidirectional, i.e., the projections from CA1 to the PreS are predominant, while few are from the PreS to CA1. Unfortunately, the present study had no cases in which BDA was injected only in the deep layers of the PreS; therefore, the possibility that projections from the PreS to CA1 could not be detected cannot be ruled out. However, in our results for the marmoset, there was no significant cluster of labeled terminals in CA1, at least in cases in which BDA was injected into the superficial layers of the PreS (as in case 09E in Figures 6, 8). Furthermore, the number of retrogradely labeled cells throughout the PreS in case 09D, in which the CTB injection site included CA1 besides the PreS, was not significantly higher than in case 09E, in which the CTB injection sites included the PreS but did not contain CA1 (data not shown). Therefore, unidirectional projection from CA1 to PreS may be significant in the marmoset, as well as in the rabbit.

Previously, two distinct sublayers composed of deep and superficial CA1 pyramidal cells have been reported and implicated in the heterogeneity of EC-CA1 connectivity (Masurkar et al., 2017; Valero and de la Prida, 2018). Mizuseki et al. (2011) showed that deep CA1 pyramidal cells are more likely to be place cells than superficial CA1 cells, and Masurkar et al. (2017) demonstrated that MEC (which provide spatial information) preferentially project to the deep part of the pyramidal cell layer of CA1, especially in the proximal part, whereas LEC (which provide non-spatial information) mainly project to the superficial part of distal CA1. In the marmoset hippocampus, the pyramidal cell layer of CA1 was distinctively wide and the cells were uniformly distributed throughout the depth of the pyramidal cell layer (Figures 1A,C). In our CTB injection results, presubicular projection cells were diffusely distributed in the pyramidal cell layer of CA1 in most cases, as seen in Figure 3. This suggests that sublaminar parcellation in marmoset CA1 is unclear from the perspective of CA1-PreS projections. The projections to the EC originating from each part of CA1 also need to be studied in greater detail to confirm and define the topographic organization of direct hippocampo-entorhinal back projections in marmosets. Our findings have implications for future investigations of the functional differentiation of the hippocampal and parahippocampal areas in the mammalian brain.

### Sublamination in the Pyramidal Cell Layer of the Sub

In this study, we found a clear sublaminar pattern in the distribution of cortical-projection cells in the marmoset Sub (Figure 9C). The population of the PreS projection cells was localized in the middle part along the superficial-to-deep axis of the pyramidal cell layer of the Sub, especially in the mid-to-distal portion, as seen in cases 09E, M27, M23, 10F, and 13H (Figures 3–5). This distribution pattern is similar to that observed in the rat and rabbit Sub (Honda and Ishizuka, 2015; Honda and Shibata, 2017). Previously, Ishizuka (2001) indicated distinct sublimation of the subicular pyramidal cell layer in the rat from an analysis of the distribution pattern of cells of origin of the subcortical projections, i.e., the efferents to the anterior thalamic nuclei, medial mammillary nucleus, and nucleus accumbens. As mentioned in the Results section, several non-labeled cells were distributed among the labeled-cell cluster of the Sub, suggesting that several subpopulations may exist in each cortical-projection cell group. Combined with an immunohistochemical approach (Ishihara et al., 2020), further analysis of the localization of subicular neurons that give rise to descending projections to each subcortical area is needed to elucidate the connectional organization of the marmoset Sub more precisely. Furthermore, it is difficult to identify the boundary between the Sub and adjacent regions (in particular, the boundary between the deepest part of the pyramidal cell layer of the Sub and the layers V-VI of the PreS) using Nissl staining. In this regard, a combination of injection experiments and immunostaining will be necessary to clarify the boundaries of the Sub.

### Intrinsic Connections Within Each Area

Our data suggest that many intrinsic neurons in the marmoset are diffusely distributed throughout the entire pyramidal cell layer of the Sub in a wide range along the rostrocaudal and deep-to-superficial axes. We infer that a wide range of associational connections is present, as previously reported for the rat Sub (Köhler, 1985). Furthermore, our previous study using rats indicated that cells in layers II and V of the PreS mostly give rise to longitudinal projections in the PreS and also confirmed massive descending projections from layer II to layer V over the entire extent of the PreS (Honda et al., 2008). The present results suggest that similar to the rat PreS, terminal regions of intrinsic projection within the marmoset PreS are concentrated in layers I-II, lamina dissecans, and layer V. Further detailed analysis of the intrinsic connections in each area will be necessary to understand the features that are characteristic of associational connections in the hippocampal and parahippocampal areas of marmosets.

### Methodological Considerations

Retrogradely labeled cells were observed in several BDA injection cases, such as the retrogradely labeled layer II cells of the EC in cases 08Q and 08R (Figure 7). We considered BDA absorption from cut fibers of perforant path projections to be an artifact and carefully checked for such false signals in other experimental cases. As a result, we were unaware of any other examples in this study. Both BDA and CTB injections can produce labeling of axon collaterals of retrogradely labeled neurons (Chen and Aston-Jones, 1998; Reiner and Honig, 2006). We also checked our results and excluded such labeling (e.g., anterograde labeling in layers I-II of the EC in cases 08Q and 08R) from the evaluation.

In some cases, CTB and/or BDA injections were performed in both hemispheres (Table 1), and we could clearly distinguish the labeling of CTB and BDA by biotin blocking and/or nickel intensification (see Materials and Methods). Nevertheless, on the side of BDA injection, some CTB-labeled cells that may project to the contralateral (i.e., the same side of CTB injection) areas overlapped with retrograde labeling of BDA around the BDA injection site. To avoid confusion, ipsilateral (associational) connectivity was mainly presented in this report. Unfortunately, in case 10F, the BDA injection was ineffective, and we only evaluated CTB labeling. Future studies should include multiple cases of microinjections confined to the PreS to elucidate the projection patterns of the PreS.

### Functional Considerations

Previous studies have reported some basic connectivities that are common across different animal species, such as the trisynaptic circuit originating from the superficial layers of the EC and the back projections originating from the hippocampal formation to the deep layers of the EC (mouse: van Groen et al., 2003; rat: Steward and Scoville, 1976; guinea pig: Sørensen and Shipley, 1979; cat: van Groen et al., 1986; Witter and Groenewegen, 1986; Ino et al., 1998, 2001; monkey: Witter and Amaral, 1991, 2021). It can be predicted that marmosets have connections similar to those in other species. Furthermore, our results reveal the presence of a large number of CA1-PreS projections (Figure 9A), which have not been reported in rodents or other non-human primates. Head direction cells have already been found in the PreS of rhesus macaques (Robertson et al., 1999) and additional presubicular connections, such as CA1-PreS projections, are expected to be involved in the characteristic function of the head direction system in the marmoset brain. Courellis et al. (2019) reported that cells in the marmoset hippocampus (analogous to the place cells in other mammalian species) encode self-positioning during free-moving exploration. However, theta oscillations weakly correlated with either place-cell activity or locomotion (Courellis et al., 2019). Additional connections via the PreS in the hippocampal-parahippocampal network can contribute to the distinct functional properties of different animal species.

As for the topographic organization in the CA1-PreS connectivity, our results suggest that cells in a small and localized CA1 region (especially in the rostral part) could project to a wide range of the PreS along the rostrocaudal and proximodistal axes (Figure 9B). It is important to note that the rostro-caudal (antero-posterior) direction of the hippocampal formation in primates corresponds to the ventro-dorsal (temporo-septal) direction of the hippocampal formation in rodents (Strange et al., 2014). Thus, if we translate our results of marmosets to rodents, we can say that the ventral (temporal) portion of CA1 tends to project over a wide range of the PreS. A common belief is that the dorsal (or posterior) CA1 is involved in spatial memory and navigation, while the ventral (or anterior) CA1 plays an important role as a component of social memory storage (Okuyama et al., 2016) and also mediates anxiety-related behaviors and contextual fear memory (Jimenez et al., 2018, 2020). It is unclear whether these features of the ventral CA1 also apply to the primate; however, at least in the marmoset, the distribution of input cells to the medial prefrontal cortex has been reported to be restricted to the rostral (as opposed to dorsal or lateral) part of the hippocampal formation, including CA1 and the hippocampal peduncle, and it is easy to predict that the characteristics of fiber communication patterns would be anatomically different in the rostral and caudal hippocampus.

In conclusion, our results, which show that a large part of the PreS receives many inputs from the rostral part of CA1, raise the possibility that the marmoset PreS may receive various kinds of information from the rostral CA1 that are not related to spatial recognition, such as the head direction signal.

## Data Availability Statement

The original contributions presented in this study are included in the article/supplementary material, further inquiries can be directed to the corresponding author.

## Ethics Statement

The animal study was reviewed and approved by the Animal Care and Use Committee of the Tokyo Metropolitan Institute of Medical Science (TMIMS) and Ehime University.

## Author Contributions

YH, YK, and KM-I: study concept and design. YH, TS, and SM: acquisition of data. YH: drafting of the article and obtained funding. SM and YK: critical revision of the article for important intellectual content. All authors had full access to all the data in the study and take responsibility for the integrity of the data and the accuracy of the data analysis.

## Conflict of Interest

The authors declare that the research was conducted in the absence of any commercial or financial relationships that could be construed as a potential conflict of interest.

## Publisher’s Note

All claims expressed in this article are solely those of the authors and do not necessarily represent those of their affiliated organizations, or those of the publisher, the editors and the reviewers. Any product that may be evaluated in this article, or claim that may be made by its manufacturer, is not guaranteed or endorsed by the publisher.

## Abbreviations

A35/36: areas 35 and 36 (the perirhinal cortex)
AB: angular bundle
BDA: biotinylated dextran amine
CA: cornu ammonis
CTB: cholera toxin-B subunit
D: distal
DAB: 3,3′-diaminobenzidine
DG: dentate gyrus
EC: entorhinal cortex
fim: fimbria
HRP: horseradish peroxidase
IS: injection site
ld: lamina dissecans
MEC: medial part of the EC
mid: middle part along the superficial-to-deep axis of the pyramidal layer of the Sub
ml: molecular layer
LEC: lateral part of the EC
P: proximal
ParS: parasubiculum
PB: phosphate buffer
PCL: pyramidal cell layer
PreS: presubiculum
rf: rhinal fissure
slm: stratum lacunosum-moleculare
so: stratum oriens
sp: stratum pyramidale
sr: stratum radiatum
Sub: subiculum
TBS: Tris-buffered saline
TF: area TF
TH: area TH.
